# Mechanobiology and Molecular Regulation of Periodontal Tissue Remodeling During Orthodontic Tooth Movement: From Periodontal Phenotype to Precision Orthodontics—A Narrative Review

**DOI:** 10.3390/ijms27177698

**Published:** 2026-08-28

**Authors:** Bartłomiej Górski, Edyta Kalina, Marcin Derwich

**Affiliations:** 1 Department of Periodontology and Oral Diseases, Medical University of Warsaw, 02-091 Warsaw, Poland; bartlomiej.gorski@wum.edu.pl; 2 Department of Orthodontics, Medical University of Warsaw, 02-091 Warsaw, Poland; edyta.kalina@wum.edu.pl; 3 Department of Pediatric Dentistry, Lodz Medical University, 90-419 Lodz, Poland

**Keywords:** orthodontic tooth movement, mechanobiology, periodontal phenotype, mechanotransduction, alveolar bone remodeling, gingival recession, molecular biomarkers, artificial intelligence, cone-beam computed tomography, precision orthodontics

## Abstract

Orthodontic tooth movement has traditionally been planned according to biomechanical principles; however, growing evidence indicates that treatment outcomes are strongly influenced by individual biological responsiveness. This narrative review summarizes current knowledge on the mechanobiological and molecular mechanisms regulating periodontal tissue remodeling during orthodontic tooth movement and discusses their implications for personalized orthodontic treatment. The literature was critically analyzed with particular emphasis on mechanotransduction pathways, including Piezo1, TRPV4, integrin-mediated signaling, Hippo-YAP/TAZ, Wnt/β-catenin, and the RANK/RANKL/OPG axis, together with inflammatory mediators, extracellular matrix remodeling, periodontal phenotype, and emerging molecular biomarkers associated with periodontal susceptibility. Recent advances in cone-beam computed tomography, intraoral optical scanning, STL-CBCT fusion, and artificial intelligence were also reviewed for their potential to improve biologically informed diagnosis and treatment planning. Current evidence indicates that periodontal complications, including gingival recession, alveolar bone loss, and root resorption, arise from complex interactions between orthodontic biomechanics and patient-specific biological characteristics rather than tooth movement alone. Integration of mechanobiology, molecular profiling, advanced imaging, and artificial intelligence provides the foundation for precision orthodontics, enabling individualized risk assessment, biologically guided treatment planning, and improved long-term periodontal stability.

## 1. Introduction

Orthodontic tooth movement is a biologically complex process involving coordinated responses of the periodontal ligament (PDL) and alveolar bone, mediated through the interconnected cascade of cellular and molecular mechanisms [1]. According to the widely accepted theory of orthodontic tooth movement, application of force to a tooth induces the formation of compression and tension zones within periodontal ligament (PDL), initiating biological responses. Bone resorption occurs in the pressure zone, whereas bone tissue apposition takes place in the tension zone.

However, orthodontic tooth movement should not be considered solely as a mechanical displacement of teeth, but rather as a highly regulated mechanobiological process governed by inflammatory signaling pathways, cellular adaptation, and individual tissue susceptibility [2,3]. Importantly, the biologic response to orthodontic forces varies substantially among individuals and depends on local anatomic, phenotypic, and systemic factors.

The alveolar contour, also referred to as alveolar envelope, represents the biological limit of orthodontic tooth movement. Displacement of the tooth root beyond the boundaries of the alveolar envelope poses a threat of bone dehiscence, fenestration and, consequently, gingival recession (GR) [4]. Among these complications, GR remains one of the most clinically relevant due to its esthetic implications, dentin hypersensitivity, increased susceptibility to root caries, non-carious cervical lesions, plaque accumulation, and patients’ concern regarding long-term tooth prognosis.

Susceptibility to periodontal complications is not homogeneous among patients. Individuals presenting with a thin periodontal phenotype are generally considered more vulnerable to adverse tissue responses during orthodontic treatment [5,6,7]. Thin gingival tissue, reduced keratinized tissue width (KTW), and a delicate alveolar bone morphotype may provide less resistance to mechanical trauma, inflammatory challenges, and excessive tooth displacement, especially beyond the alveolar envelope.

Over the years, orthodontic treatment planning has primarily focused on achieving ideal occlusal relationships, facial esthetics, and functional harmony of the stomatognathic system. However, increasing awareness of orthodontic-related periodontal interactions, combined with growing esthetic expectations and advances in digital diagnostics, has shifted attention toward biologically guided treatment planning.

Contemporary orthodontics increasingly emphasizes individualized treatment strategies based on the patient’s gingival phenotype, alveolar bone morphotype, and anatomical limits of tooth movement. Recent advances in cone-beam computed tomography (CBCT), intraoral optical scanning, STL-CBCT fusion, and artificial intelligence (AI) offer unprecedented opportunities for personalized risk assessment and prediction of feasible periodontal complications before treatment initiation.

Therefore, the aim of this narrative review is to summarize the current evidence regarding the molecular and biological mechanisms underlying periodontal complications during orthodontic tooth movement and to discuss how periodontal phenotype assessment, advanced imaging, digital workflows, and AI may contribute to precision-oriented orthodontic treatment planning and individualized risk assessment.

## 2. Literature Search Strategy

This narrative review was informed by a literature search conducted in PubMed/MEDLINE, Scopus, and Web of Science for articles published from database inception to June 2026. The search strategy combined terms related to orthodontic tooth movement and periodontal remodeling with terms addressing mechanobiology, molecular signaling, periodontal phenotype, biomarkers, imaging, and precision orthodontics. Principal search terms included “orthodontic tooth movement”, “periodontal remodeling”, “mechanobiology”, “mechanotransduction”, “Piezo1”, “TRPV4”, “integrin”, “FAK”, “YAP”, “TAZ”, “Hippo pathway”, “Wnt/β-catenin”, “RANK”, “RANKL”, “OPG”, “periodontal phenotype”, “gingival recession”, “alveolar bone”, “gingival crevicular fluid”, “biomarkers”, “CBCT”, “intraoral scanning”, “artificial intelligence”, and “precision orthodontics”, used individually and in relevant combinations.

Original research articles, systematic reviews, meta-analyses, and relevant narrative reviews published in English were considered for inclusion when they addressed the biological or molecular mechanisms of orthodontic tooth movement, periodontal tissue response and complications, periodontal phenotype, molecular biomarkers, imaging-based assessment, or emerging approaches to individualized orthodontic risk assessment. Both human studies and relevant in vitro and animal studies were considered, particularly for mechanistic pathways for which direct clinical evidence remains limited. Priority was given to recent studies and higher-level clinical evidence where available, while seminal experimental studies were retained when necessary to describe established or emerging biological mechanisms. Articles not directly relevant to the scope of the review, publications for which sufficient methodological information was unavailable, and duplicate records were excluded. Reference lists of relevant articles were additionally screened to identify pertinent publications not retrieved through the primary database search. No restriction on the initial publication date was applied, and the search was updated through June 2026. Given the narrative nature of the review, study selection was based on relevance to the predefined thematic scope rather than on a formal systematic-review protocol.

## 3. Biological Basis of Periodontal Complications During Orthodontic Tooth Movement

### 3.1. Mechanobiology of Orthodontic Tooth Movement

Biomechanics in orthodontics refers to the interaction between externally applied mechanical forces and the biological response of periodontal tissues. Consequently, orthodontic tooth movement should be regarded as a process of mechanobiological adaptation, in which mechanical stimuli are converted into intracellular biochemical signals regulating tissue remodeling. This phenomenon, referred to as mechanotransduction, involves coordinated responses of PDL fibroblasts, osteocytes, osteoblasts, osteoclasts, vascular cells, and immune cells [8]. The direction, vector, and point of force application determine the location of pressure and tension zones within PDL, whereas the magnitude and duration of the applied force influence the local vascular and cellular inflammatory response. Planning tooth movement requires estimation of the tooth’s center of resistance. In single-rooted teeth, the center of resistance is located approximately one-third of the root length apical to the alveolar crest, whereas in multirooted teeth the center of resistance is situated in the region of the root furcation. However, the position of the center of resistance varies depending on the level of alveolar bone support, root length and morphology, the characteristics of the alveolar bone, particularly bone thickness; therefore, its position may change dynamically during orthodontic treatment [9].

Importantly, the biologic response to orthodontic forces is highly individualized and depends not only on force itself but also on the patient’s periodontal phenotype, alveolar bone morphology, vascularization, and inflammatory responsiveness. Consequently, identical orthodontic forces may induce markedly distinct tissue responses among different patients, potentially explaining interindividual variability in susceptibility to periodontal complications.

### 3.2. Mechanosensitive Signaling Pathways in Orthodontic Tooth Movement

Mechanical forces generated during orthodontic treatment are initially detected by specialized mechanosensitive structures expressed by PDL fibroblasts, osteocytes, osteoblasts, mesenchymal stem cells (MSCs), and vascular endothelial cells. These mechanosensors convert extracellular mechanical stimuli into intracellular biochemical signals through the process of mechanotransduction, thereby regulating inflammatory responses, extracellular matrix remodeling, osteogenesis, and alveolar bone adaptation [8,10]. It should be emphasized that the evidence supporting individual mechanotransduction pathways during orthodontic tooth movement is derived from different experimental levels. Much of the detailed mechanistic evidence concerning Piezo1, TRPV4, integrin-FAK, YAP/TAZ, and Wnt/β-catenin signaling originates from in vitro studies and preclinical animal models. Human clinical evidence is comparatively limited and is derived primarily from analyses of gingival crevicular fluid (GCF)and other biomarkers, imaging studies, and observational assessments of periodontal tissue responses. Therefore, the molecular interactions described below should be interpreted as an evolving mechanistic framework rather than as fully established clinical pathways in humans.

Among the currently identified mechanosensors, the mechanically activated ion channel Piezo1 has emerged as a central regulator of periodontal mechanobiology. Mechanical deformation of the plasma membrane induces Piezo1 opening, resulting in rapid Ca^2+^ influx and activation of downstream signaling pathways involved in osteoblast differentiation, osteocyte survival, angiogenesis, and osteoclastogenesis. Piezo1 signaling promotes the expression of osteogenic markers, including Runt-related transcription factor 2 (Runx2), alkaline phosphatase (ALP), and osteocalcin, while simultaneously modulating the receptor activator of nuclear factor κB/receptor activator of nuclear factor κB ligand/osteoprotegerin (RANK/RANKL/OPG) axis, thereby coordinating the balance between bone formation and resorption. Experimental studies have further demonstrated that impaired Piezo1 signaling compromises alveolar bone remodeling and reduces the efficiency of orthodontic tooth movement [9,11,12].

Another important mechanosensitive ion channel is transient receptor potential vanilloid 4 (TRPV4), which responds to mechanical stretch, fluid shear stress, and osmotic changes. Similar to Piezo1, TRPV4 regulates intracellular calcium homeostasis and modulates inflammatory signaling, prostaglandin synthesis, and osteoblast differentiation. Experimental evidence suggests that coordinated activation of Piezo1 and TRPV4 contributes to the early cellular response to orthodontic loading and facilitates physiologic bone remodeling [10,11].

Mechanical forces are also transmitted through integrin receptors, which establish a structural connection between extracellular matrix proteins and the intracellular actin cytoskeleton. Mechanical activation of integrins promotes phosphorylation of focal adhesion kinase (FAK), initiating multiple downstream signaling cascades, including mitogen-activated protein kinase/extracellular signal-regulated kinase (MAPK/ERK), phosphoinositide 3-kinase/protein kinase B (PI3K/AKT), and ras homolog family member A/rho-associated coiled-coil containing protein kinase (RhoA/ROCK) pathways. These signaling networks regulate cytoskeletal organization, focal adhesion turnover, cell migration, and extracellular matrix remodeling, enabling periodontal tissues to continuously adapt to changing biomechanical conditions. Importantly, integrin-focal adhesion kinase (FAK) signaling also facilitates nuclear localization of the mechanosensitive transcriptional co-activators: Yes-associated protein (YAP) and transcriptional co-activator with PDZ-binding motif (TAZ), thereby linking extracellular mechanical forces with transcriptional regulation [3,12].

Among intracellular mechanotransduction pathways, the Hippo-YAP/TAZ signaling axis has recently received considerable attention [13]. Under conditions of low mechanical loading, Hippo kinase activity retains YAP and TAZ within the cytoplasm, limiting their transcriptional activity. In contrast, mechanical stimulation suppresses Hippo signaling, allowing YAP/TAZ to translocate into the nucleus, where they interact with TEA domain transcription factors (TEAD) and activate genes regulating cell proliferation, extracellular matrix synthesis, angiogenesis, and osteogenic differentiation. Increasing evidence indicates that YAP/TAZ serve as central molecular integrators of mechanical loading within the PDL and alveolar bone during orthodontic tooth movement [12,14].

The canonical wingless-related integration site/beta-catenin (Wnt/β-catenin) signaling pathway represents another essential regulator of mechanically induced bone formation. Mechanical stimulation promotes stabilization and nuclear accumulation of β-catenin, enhancing osteoblast differentiation through activation of osteogenic transcription factors, particularly Runx2. This pathway is tightly regulated by sclerostin (SOST), a glycoprotein secreted predominantly by osteocytes that inhibits Wnt signaling by binding to the low-density lipoprotein receptor-related protein 5/6 (LRP5/6) receptor complex. Site-specific modulation of SOST expression during orthodontic loading is considered an important mechanism controlling the balance between bone apposition and bone resorption, thereby contributing to adaptive remodeling of the alveolar process [15,16].

Emerging evidence also indicates that Notch signaling contributes to periodontal tissue adaptation by regulating MSC differentiation, osteoblast maturation, angiogenesis, and immune responses. Extensive crosstalk between Notch, Wnt/β-catenin, and Hippo–YAP/TAZ pathways coordinates cellular communication during alveolar bone remodeling and supports maintenance of periodontal homeostasis under mechanical loading [10,14].

**Figure 1:**
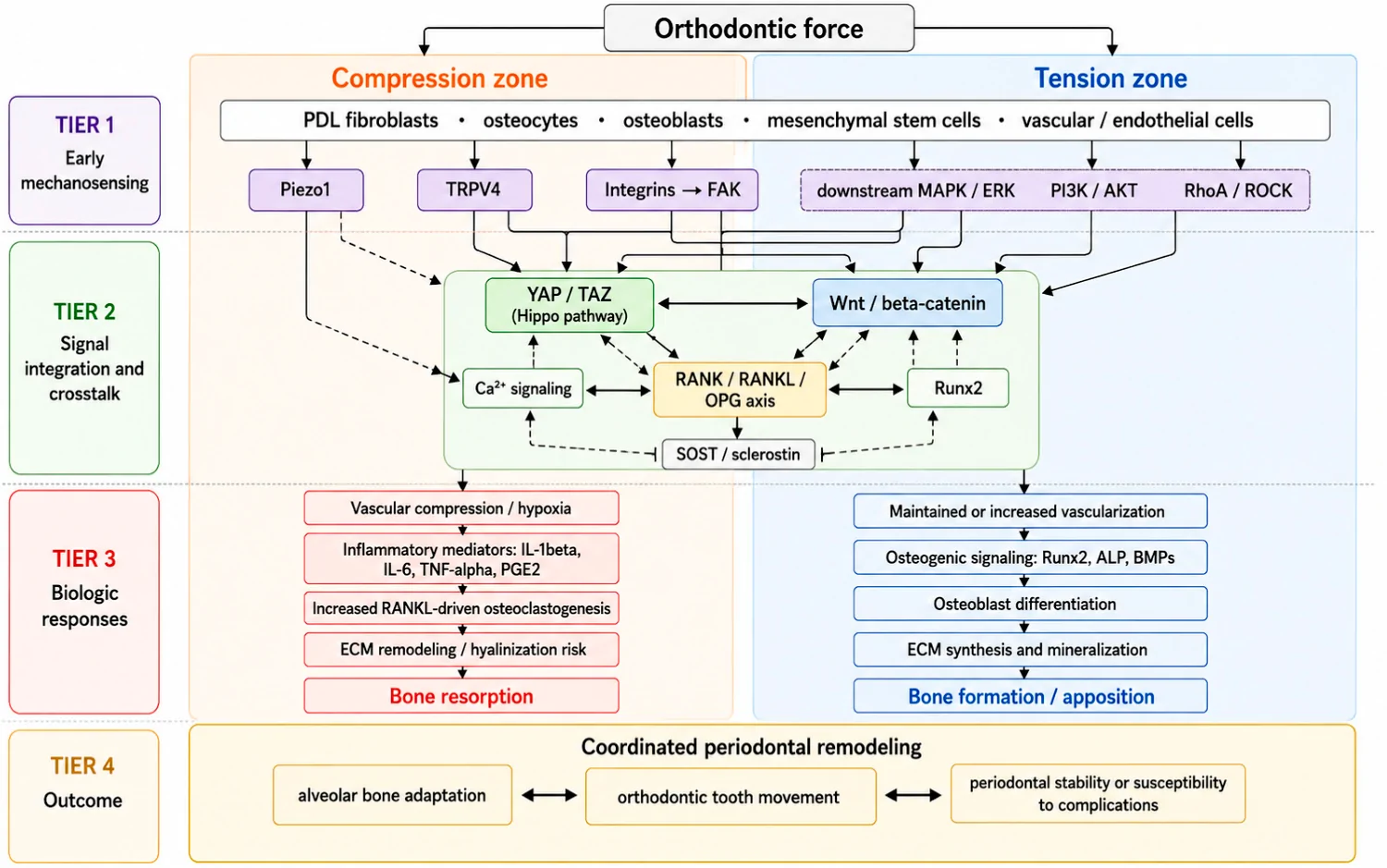
Integrated mechanobiological signaling during orthodontic tooth movement. Mechanical loading is sensed by PDL fibroblasts, osteocytes, osteoblasts, MSCs, and vascular cells through mechanosensitive ion channels (Piezo1 and TRPV4) and integrin–FAK signaling. Crosstalk with YAP/TAZ, Wnt/β-catenin, and RANK/RANKL/OPG pathways coordinates inflammatory responses, extracellular matrix remodeling, osteoclastogenesis in compression-dominant regions, and osteogenesis in tension-dominant regions. The relative contribution of these pathways varies according to cell type, spatial location, force characteristics, and stage of orthodontic tooth movement. Much of the detailed mechanistic framework is currently based on in vitro and preclinical evidence, with more limited direct validation in humans.

Collectively, these mechanosensitive pathways form an integrated signaling network linking externally applied orthodontic forces with intracellular responses regulating inflammation, extracellular matrix remodeling, osteogenesis, osteoclastogenesis, and angiogenesis. Importantly, activation of these pathways is influenced not only by the magnitude, direction, and duration of orthodontic forces but also by patient-specific biologic characteristics, including periodontal phenotype, osteogenic potential, vascularization, and systemic health. This complex mechanobiological network provides a molecular explanation for the marked interindividual variability in periodontal adaptation and susceptibility to complications, such as alveolar bone loss, root resorption, and GR observed during orthodontic treatment. Table 1 presents molecular pathways regulating orthodontic tooth movement. Importantly, these pathways should not be viewed as independent linear cascades. Rather, available experimental evidence suggests extensive spatial and temporal crosstalk among mechanosensing, inflammatory signaling, osteoclastogenesis, and osteogenesis, with their relative contribution varying between compression and tension zones, among different periodontal cell populations, and across successive stages of orthodontic tooth movement (Figure 1).

### 3.3. Periodontal Ligament Response to Orthodontic Forces

In the pressure zone, narrowing of PDL space occurs, accompanied by compression of blood vessels, reduced oxygen tension, and increased carbon dioxide concentration. As a result, the PDL fiber architecture is disrupted. A compressive strain was found to decrease collagen I mRNA, type I collagen, and increase matrix metalloproteinase-2 (MMP2) mRNA levels. Simultaneously, new fibers composed of type III collagen are formed to maintain the attachment [17].

The compression induces strain and microdamage to the bone matrix, leading to osteocyte apoptosis. Recent evidence indicates that osteocytes may play a pivotal role in the mechanosensory response to orthodontic loading. Mechanical strain activates intracellular signaling pathways involving SOST, YAP/TAZ transcriptional regulators, integrins, and mechanosensitive ion channels such as Piezo1, thereby modulating osteoclastogenesis, extracellular matrix remodeling, and bone adaptation [16]. Metalloproteinases produced by osteoblasts degrade osteoid. Dysregulation of these pathways may contribute to unfavorable periodontal responses, particularly in anatomically vulnerable sites characterized by thin alveolar bone morphology. An increased number of osteoclasts appears along the surface of the alveolar bone trabeculae, leading to bone resorption. When the applied orthodontic force exceeds 20–25 g/cm^2^ of root surface area, sterile necrosis (hyalinization) of PDL may develop [18].

In the tension zone, PDL space widens, and blood vessels exhibit increased luminal diameter, resulting in elevated oxygen levels and reduced carbon dioxide concentration. The PDL fibers built from collagen type I maintain an organized arrangement. A new extracellular matrix is formed and then mineralized. In an in vitro study, human PDL cells in response to tensile strains increasingly expressed osterix, bone sialoprotein, ALP, type I collagen, and bone morphogenetic proteins (BMPs), all of which promote bone formation and tissue remodeling [15].

The process of bone apposition proceeds more slowly than tissue resorption, which explains the presence of an enlarged PDL space during orthodontic tooth movement. This biologic imbalance between resorptive and appositional processes may be clinically relevant. In patients with a thin periodontal phenotype, insufficient compensatory bone formation may predispose to dehiscence formation and subsequent GR.

### 3.4. Inflammatory Signaling Pathways (RANK/RANKL/OPG, Cytokines, Osteoclastogenesis)

Orthodontic tooth movement is mediated by a tightly regulated inflammatory cascade that resembles sterile inflammation. Mechanical loading induces deformation of the extracellular matrix and vascular alterations within PDL, resulting in the release of inflammatory mediators that orchestrate bone resorption and formation [19]. During the initial phase, the inflammatory reaction is acute and characterized by transmigration of leukocytes through the vascular endothelium and increased release of inflammatory mediators that stimulate bone remodeling. In the early phase of force application, tooth displacement occurs immediately as a result of movement within PDL space. Subsequently, tooth movement becomes markedly reduced or temporarily arrested until macrophages, multinucleated giant cells, and osteoclasts eliminate hyalinized PDL fibers and necrotic tissue. Tooth movement generally resumes after approximately 20–30 days [9].

However, orthodontic tooth movement should not be interpreted as a sequence of isolated resorptive and appositional events. Contemporary evidence suggests that bone resorption and bone formation occur simultaneously and continuously throughout treatment, with their relative predominance varying depending on local biomechanical and biologic conditions [2]. The magnitude and balance of this inflammatory response may partially explain interindividual differences in susceptibility to periodontal complications during orthodontic treatment.

Mediators involved in bone resorption include interleukin-1β (IL-1β), interleukin-6 (IL-6), interleukin-11 (IL-11), prostaglandins, receptor activator of nuclear factor κB ligand (RANKL), tumor necrosis factor-α (TNF-α), parathyroid hormone (PTH), and macrophage colony-stimulating factor (M-CSF). Factors associated with osteogenesis and bone apposition include core-binding factor alpha 1 (Cbfa1/Runx2), transforming growth factor-β (TGF-β), BMPs, androgens, and growth hormone (GH) [10]. After approximately two days, the acute inflammatory response progresses into a chronic phase characterized by the presence of fibroblasts, osteoblasts, and endothelial cells within the periodontal tissues surrounding the teeth. Chronic inflammation persists until subsequent appliance activation or gradually subsides several weeks after the cessation of orthodontic force application [20].

The RANK/RANKL/OPG signaling axis is considered the principal molecular pathway regulating osteoclast differentiation, activation, and survival during orthodontic tooth movement. Increased expression of RANKL is stimulated by inflammatory cytokines, prostaglandin E2 (PGE2), hypoxia, calcitriol, parathyroid hormone, and macrophage colony-stimulating factor (M-CSF). Binding of RANKL to its receptor RANK on osteoclast precursor cells promotes osteoclastogenesis and bone resorption. Conversely, osteoprotegerin (OPG), a soluble glycoprotein acting as a decoy receptor, inhibits osteoclast differentiation by preventing RANKL-RANK interaction, thereby exerting a protective effect against excessive bone resorption [21]. Experimental evidence further supports the critical role of this pathway in orthodontic remodeling. In mice lacking RANKL expression, both osteoclast formation and orthodontic tooth movement were significantly impaired, confirming that RANKL signaling is indispensable for alveolar bone adaptation to orthodontic loading [22]. Table 2 outlines molecular biomarkers associated with periodontal remodeling.

Importantly, inflammatory responses to orthodontic forces are further modulated by patient-related systemic factors. Diseases such as diabetes mellitus, osteoporosis, autoimmune and endocrine disorders, cardiovascular diseases, and chronic kidney diseases may alter bone metabolism and periodontal tissue remodeling, thereby affecting orthodontic treatment outcomes [23,24]. Likewise, medications including bisphosphonates and nonsteroidal anti-inflammatory drugs (NSAIDs) may inhibit orthodontic tooth movement, whereas glucocorticosteroids have been associated with accelerated tissue remodeling [25,26]. Interestingly, in perimenopausal women, declining estrogen levels were associated with reduced osteoblastic activity and increased expression of receptor activator of nuclear factor κB ligand (RANKL), thereby enhancing osteoclastic differentiation and bone resorption [24]. Furthermore, nicotine exposure has been shown to modulate inflammatory pathways within periodontal tissues, including increased expression of interleukin-1β (IL-1β), potentially amplifying tissue susceptibility to negative bone remodeling [27].

Taken together, orthodontic tooth movement is governed by a highly dynamic inflammatory network in which local mechanobiological responses interact with systemic and patient-specific factors. Differences in these biologic responses may partly explain why periodontal changes, such as GR and alveolar bone loss, occur in some patients despite apparently similar orthodontic mechanics.

### 3.5. Alveolar Bone Remodeling and Cortical Bone Adaptation

Orthodontic tooth movement should remain within the biologic limits of the alveolar housing, whose adaptive capacity determines the extent to which teeth may be displaced without compromising periodontal integrity. Orthodontic force-induced alveolar bone remodeling is a highly dynamic process regulated by coordinated mechanobiological signaling, inflammatory response, and continuous adaptation of mineralized and soft tissues to mechanical loading [28].

Emerging evidence supports the existence of a functional PDL–periosteum axis, which plays an important role in cortical bone adaptation during orthodontic treatment. The periosteum, together with PDL cells, contains MSCs capable of differentiating into osteoblasts and other connective tissue cells. Mechanical stimulation activates multiple mechanotransduction pathways, including Runx2, YAP/TAZ, integrin-mediated signaling, and prostaglandin-dependent pathways, thereby regulating extracellular matrix remodeling, osteogenesis, angiogenesis, and inflammatory response. Activated progenitor cells release numerous bioactive mediators, including PGE2, IL-6, TNF-α, and vascular endothelial growth factor (VEGF), which coordinate alveolar bone remodeling and vascular adaptation during orthodontic tooth movement [12,29].

Nevertheless, the adaptive capacity of alveolar bone is finite. Cortical bone remodeling depends on multiple patient-specific factors, including alveolar bone thickness, vascularization, osteogenic potential, periodontal phenotype, systemic health, and the magnitude and duration of orthodontic forces. When tooth movement exceeds this biologic capacity, compensatory bone apposition may become insufficient to preserve cortical integrity, increasing the likelihood of alveolar dehiscence and subsequent GR.

The pattern of alveolar bone remodeling is strongly influenced by the type of orthodontic tooth movement. During uncontrolled tipping, compressive stresses are concentrated at the cervical aspect of the alveolar bone, whereas tensile stresses develop near the root apex, producing non-uniform mechanical loading and localized cortical remodeling [30,31]. CBCT studies have consistently demonstrated that excessive labial proclination is associated with reduced facial alveolar bone thickness, apical displacement of the alveolar crest, and increased cementoenamel junction (CEJ)-to-alveolar crest distance on both the labial and lingual aspects of the alveolar process [32,33,34,35,36].

Longitudinal CBCT investigations further indicate that alveolar dehiscences are common even before orthodontic treatment and increase substantially following excessive incisor proclination. Matsumoto et al. reported that the prevalence of dehiscences affecting mandibular incisors increased from 32% to 58% in male patients and from 24% to 45% in female patients after treatment, with approximately 8° of lower incisor proclination corresponding to a 50% probability of approximately 2 mm of vertical alveolar bone loss [37]. These observations emphasize that excessive labial tooth movement may exceed the adaptive capacity of thin cortical plates, particularly in patients with thin periodontal phenotype or pre-existing osseous defects.

In contrast, controlled bodily movement and adequate root torque distribute mechanical stresses more uniformly along the root surface, thereby reducing localized cortical overload and promoting more physiologic bone remodeling. Appropriate biomechanical control may therefore preserve alveolar bone morphology and reduce the risk of periodontal complications associated with excessive tooth displacement [38].

Different patterns of bone adaptation are also observed during vertical tooth movement. Intrusion primarily concentrates mechanical stresses in the apical PDL, increasing the risk of orthodontically induced external apical root resorption while producing relatively limited remodeling of the alveolar crest [39]. Conversely, slow orthodontic extrusion (<1 mm/month) promotes coordinated coronal migration of alveolar bone and gingival tissues, maintaining the physiologic relationship between the CEJ, alveolar crest, and gingival margin. Rapid extrusion, however, exceeds the regenerative capacity of the periodontium, resulting in tooth movement without proportional coronal bone apposition and consequently increasing the CEJ-to-alveolar crest distance [40,41].

The magnitude and duration of orthodontic forces further influence the biologic response of periodontal tissues. Light continuous forces maintain PDL vascularization, promote frontal bone resorption, and facilitate coordinated osteoclastic and osteoblastic activity through controlled release of cytokines, prostaglandins, and growth factors. In contrast, excessive forces produce vascular compression, ischemia, and hyalinization of the PDL, delaying tooth movement through undermining bone resorption while increasing the risk of root resorption and periodontal tissue damage [42,43]. Experimental and clinical evidence suggests that forces within the range of approximately 50–100 cN may provide favorable conditions for orthodontic tooth movement in selected clinical situations while minimizing undesirable side effects [44]. However, the appropriate force magnitude is not universal and depends on the tooth involved, root surface area, type and direction of tooth movement, force distribution, treatment mechanics, and the condition of the supporting periodontal tissues.

Overall, successful orthodontic tooth movement depends on the balance between mechanical loading and the adaptive capacity of the supporting periodontal tissues. When tooth displacement exceeds the anatomical and biological limits of the alveolar housing, the risk of dehiscence, GR, and other periodontal complications increases. Table 3 presents the biological events in periodontal tissue during orthodontic tooth movement.

### 3.6. Soft Tissue Phenotype and Gingival Stability

Periodontal soft tissue stability during orthodontic treatment is determined by the interplay among periodontal phenotype, alveolar bone morphology, tooth position, host inflammatory response, and orthodontic biomechanics.

Among these determinants, the periodontal phenotype—particularly gingival thickness (GT) and KTW—plays a pivotal role in maintaining periodontal soft tissue integrity during orthodontic treatment. Thin gingival tissues may provide less structural and vascular reserve during orthodontic loading and frequently coexist with thin facial cortical bone, increased scalloping, and greater susceptibility to dehiscence formation. Consequently, soft-tissue stability should not be considered independently from the underlying osseous morphology; rather, periodontal resilience reflects the combined adaptive capacity of the soft and hard periodontal tissues [45].

Gingival phenotype and alveolar bone morphology are closely interrelated, and reduced soft tissue thickness frequently coexists with thin cortical bone plates, increased scalloping, and higher susceptibility to dehiscence formation [46].

### 3.7. Biological Mechanisms of Gingival Recession Development

GR during orthodontic treatment is a multifactorial complication arising from the interaction between tooth movement, alveolar bone adaptation, periodontal phenotype, and individual biological susceptibility [47]. Excessive labial or lingual root displacement may reduce alveolar bone support and promote dehiscence formation, particularly at anatomically vulnerable sites with limited cortical dimensions [36,48,49,50,51]. Thin buccal cortical bone may further increase susceptibility because of its limited osteogenic potential and reduced capacity for compensatory bone apposition during orthodontic tooth movement [52,53,54]. In addition, displacement of the root toward or beyond the cortical boundary may alter the functional relationship between the PDL and periosteum, further compromising periodontal support and contributing to the development of dehiscence and subsequent GR [55]. However, orthodontic tooth movement alone is insufficient to explain recession development, as apparently similar mechanics may produce markedly different periodontal responses among patients.

Recent molecular studies further support the concept of individualized biologic susceptibility. A comprehensive bioinformatic analysis of GCF identified a core network of biomarkers involved in orthodontic tooth movement, including IL-1β, IL-6, TNF-α, PGE2, RANKL, OPG, TGF-β1, ALP, MMP-1, MMP-3, MMP-8, MMP-9, and leptin, highlighting the coordinated regulation of inflammation, extracellular matrix remodeling, and bone turnover throughout different phases of tooth movement [56]. Complementary proteomic analysis demonstrated that patients who developed GR exhibited a distinct molecular profile, with IQGAP1, ACTN1, TLN1, VASP, FN1, FERMT3, MYO1C, and RALA emerging as key proteins involved in cytoskeletal organization, cell adhesion, and mechanotransduction [57]. Together, these findings suggest that periodontal complications during orthodontic treatment are influenced not only by orthodontic biomechanics but also by patient-specific molecular responsiveness. Future risk assessment strategies may therefore integrate periodontal phenotype, three-dimensional imaging, and molecular biomarkers to improve prediction of GR before orthodontic treatment initiation.

## 4. Molecular Biomarkers of Susceptibility

Recent advances in molecular biology have considerably improved our understanding of the biological mechanisms underlying periodontal adaptation during orthodontic tooth movement. Increasing evidence indicates that interindividual variability in periodontal response is determined not only by anatomical and biomechanical factors but also by patient-specific molecular signatures reflecting inflammation, extracellular matrix remodeling, mechanotransduction, and tissue regenerative potential. Consequently, the identification of molecular biomarkers may facilitate early recognition of patients at increased risk of periodontal complications and support the development of precision-based orthodontic treatment strategies.

### 4.1. Inflammatory Biomarkers

Orthodontic tooth movement induces a sterile inflammatory response characterized by the release of numerous cytokines and inflammatory mediators into the GCF. Among these, IL-1β, IL-6, TNF-α, PGE2, RANKL, and OPG are the most extensively investigated biomarkers.

IL-1β and TNF-α are considered early mediators of periodontal inflammation, stimulating osteoclast differentiation and enhancing RANKL expression. IL-6 further amplifies inflammatory signaling and promotes osteoclastogenesis through activation of the Janus kinase/signal transducer and activator of transcription (JAK/STAT) pathway. PGE2 contributes to vasodilation, increased vascular permeability, and bone resorption, whereas the balance between RANKL and its decoy receptor OPG largely determines the rate of osteoclast activation and alveolar bone remodeling. Elevated concentrations of these mediators in GCF have consistently been associated with active orthodontic tooth movement and increased bone turnover, suggesting their potential value for monitoring tissue response during treatment [10,58].

### 4.2. Biomarkers of Extracellular Matrix Remodeling

Remodeling of the extracellular matrix represents another fundamental component of periodontal adaptation. Members of the matrix metalloproteinase family, particularly MMP-1, MMP-3, MMP-8, and MMP-9, play essential roles in collagen degradation and connective tissue remodeling during orthodontic tooth movement.

MMP-1 primarily degrades interstitial collagen, whereas MMP-3 activates multiple matrix metalloproteinases and participates in extracellular matrix turnover. MMP-8 and MMP-9 have been consistently associated with collagen degradation, inflammatory tissue destruction, and periodontal remodeling. Their activity is tightly regulated by tissue inhibitors of metalloproteinases (TIMPs), which maintain the physiological balance between matrix degradation and repair. Disturbance of the MMP/TIMP equilibrium may contribute to excessive connective tissue breakdown and increased susceptibility to GR.

Another important extracellular matrix protein is fibronectin (FN1), which mediates cell adhesion, migration, and matrix organization. Altered fibronectin expression has been associated with impaired periodontal wound healing and adaptive tissue remodeling during orthodontic treatment [57,58].

### 4.3. Mechanotransduction-Associated Biomarkers

Recent proteomic studies have identified several proteins involved in cytoskeletal organization and mechanotransduction that may represent novel biomarkers of periodontal susceptibility. Proteomic analysis of GCF demonstrated that patients developing GR during orthodontic treatment exhibit a distinct molecular profile characterized by altered expression of IQ motif containing GTPase activating protein 1 (IQGAP1), actinin alpha 1 (ACTN1), talin 1 (TLN1), vasodilator-stimulated phosphoprotein (VASP), fermitin family member 3 (FERMT3), myosin IC (MYO1C), and RAS-like proto-oncogene A (RALA) [57].

These proteins participate in cytoskeletal remodeling, focal adhesion dynamics, cell migration, mechanosensing, and force transmission between the extracellular matrix and intracellular signaling pathways. IQGAP1 functions as a scaffold protein coordinating actin cytoskeleton organization, whereas ACTN1 and TLN1 (talin-1) regulate focal adhesion assembly and integrin-mediated mechanotransduction. VASP controls actin filament polymerization, FERMT3 (Kindlin-3) stabilizes integrin activation, and MYO1C participates in intracellular force transmission and vesicular transport. Together, these proteins constitute an interconnected mechanobiological network that may determine the adaptive capacity of periodontal tissues under orthodontic loading.

### 4.4. Emerging Biomarkers and Future Perspectives

Rapid advances in multi-omics technologies are expanding the spectrum of molecular biomarkers that may improve personalized risk assessment in orthodontics. However, multi-omics-based risk prediction remains investigational and has not yet been prospectively validated for routine clinical decision-making in orthodontics. Beyond conventional inflammatory mediators, increasing attention has been directed toward microRNAs (miRNAs), extracellular vesicles (EVs), exosomes, proteomic signatures, and salivary biomarkers, which collectively provide a more comprehensive characterization of periodontal tissue adaptation and regenerative capacity.

Among these emerging biomarkers, microRNAs (miRNAs) have attracted considerable interest because they act as post-transcriptional regulators of gene expression involved in osteogenesis, osteoclastogenesis, angiogenesis, extracellular matrix remodeling, and immune response. Several miRNAs have been shown to regulate key signaling pathways associated with orthodontic tooth movement, including the RANK/RANKL/OPG axis, Wnt/β-catenin signaling, and osteogenic transcription factors such as Runx2. Differential expression of miRNAs in GCF, saliva, and PDL cells has been associated with variations in bone remodeling and inflammatory response, suggesting their potential use as predictive biomarkers of individual biologic responsiveness to orthodontic forces [10,12].

Another rapidly evolving field involves extracellular vesicles (EVs), particularly MSC-derived exosomes (MSC-Exos). Exosomes are nano-sized membrane vesicles that transport proteins, lipids, messenger RNAs, microRNAs, and other regulatory molecules between cells, thereby mediating intercellular communication. Experimental studies have demonstrated that MSC-derived exosomes promote osteoblast differentiation, suppress osteoclast activity, enhance PDL regeneration, stimulate angiogenesis, and modulate macrophage polarization toward a pro-regenerative phenotype. These biological properties make exosomes attractive not only as diagnostic biomarkers but also as potential therapeutic agents capable of enhancing periodontal regeneration, accelerating tissue adaptation, and improving post-orthodontic tooth stability. The combination of exosome-based therapies with biomaterial carriers such as injectable hydrogels may further improve local delivery and controlled release of regenerative factors [59]. Nevertheless, current evidence for exosome-based interventions in orthodontics is predominantly preclinical, and these approaches should be regarded as investigational rather than clinically established therapies.

Recent advances in proteomics have further demonstrated that comprehensive protein profiling of GCF can identify patient-specific molecular signatures associated with orthodontic tooth movement and susceptibility to periodontal complications. Unlike conventional single-biomarker approaches, proteomic analyses simultaneously evaluate hundreds of proteins involved in inflammation, extracellular matrix remodeling, mechanotransduction, angiogenesis, and bone metabolism, allowing identification of complex biological networks underlying tissue adaptation. Such molecular profiling may ultimately facilitate individualized prediction of treatment outcomes and early identification of patients at increased risk of GR or alveolar bone loss [57,58].

Although saliva represents an attractive diagnostic fluid because of its non-invasive and easily repeatable collection, its molecular composition reflects both local and systemic biological processes. In contrast, GCF originates directly from the periodontal microenvironment and more accurately reflects molecular events occurring within the PDL and surrounding alveolar bone. Consequently, GCF represents a particularly informative, site-specific source of biomarkers for monitoring orthodontic tissue remodeling and periodontal adaptation, as it reflects molecular events within the local periodontal microenvironment.

Future multimodal approaches combining molecular profiling with clinical and imaging data may improve patient-specific prediction of periodontal responses to orthodontic treatment. Prospective validation will be required before such biomarkers can be incorporated into routine clinical risk assessment.

## 5. Periodontal Phenotype as a Risk Factor

### 5.1. Definition and Components of Periodontal Phenotype

The phenotype is defined as the set of observable characteristics resulting from the interaction between genetic determinants and environmental influences. The periodontal phenotype comprises both the gingival phenotype and the osseous morphotype, together determining the biologic environment in which orthodontic tooth movement occurs. Assessment of the gingival phenotype includes GT and KTW, whereas the osseous morphotype is characterized by alveolar bone thickness and the presence of dehiscences or fenestrations [60,61].

Because alveolar bone morphology defines the biologic limits of orthodontic tooth movement, evaluation of the osseous morphotype is particularly important before treatment initiation [62,63]. The facial alveolar plate is generally thinner in the anterior region, especially around incisors and canines, is thinner in males, and tends to decrease with age [64]. Dehiscence is characterized by the absence of alveolar bone over the coronal portion of the root surface, whereas fenestration represents a localized cortical bone defect that does not involve the alveolar crest. CBCT studies have demonstrated substantial interindividual variability in alveolar bone thickness and the prevalence of pre-existing osseous defects, many of which remain clinically undetectable before orthodontic treatment [65]. In untreated individuals, the prevalence of labial fenestrations and dehiscences has been reported to be approximately 36% and 20%, respectively, with fenestrations occurring most frequently around maxillary canines [66].

Ochsenbein and Ross originally described two periodontal phenotypes—thin and thick—based on characteristic gingival, dental, and osseous morphology [67]. The thin phenotype is characterized by delicate translucent gingiva (<1 mm), a narrow band of keratinized tissue, pronounced gingival scalloping, and slender interdental papillae. In contrast, the thick phenotype presents with dense fibrotic gingiva, a wide zone of keratinized tissue, flatter gingival architecture, and broader interdental papillae. Subsequently, De Rouck et al. expanded this concept by distinguishing three phenotypes: thin-scalloped (A1), thick-scalloped (A2), and thick-flat (B) [68].

The gingival phenotype is closely associated with the underlying osseous morphotype. Thin gingival tissues are frequently accompanied by a thin facial cortical plate, increased root prominence, and a higher prevalence of dehiscences and fenestrations, whereas thick gingival tissues are generally supported by a wider and denser alveolar bone envelope, providing greater structural support during orthodontic tooth movement [69].

Periodontal phenotype represents a biologic continuum rather than a set of discrete categories. Therefore, quantitative assessment of gingival thickness, keratinized tissue width, alveolar bone dimensions, and pre-existing osseous defects may be more informative for orthodontic risk assessment than phenotype classification alone [70,71,72,73].

Orthodontic tooth movement is not inherently detrimental to periodontal tissues, but its biologic consequences depend on movement direction, periodontal phenotype, and alveolar anatomy [74].

The role of KTW remains controversial. Although reduced KTW alone does not inevitably lead to periodontal breakdown, limited keratinized tissue combined with a thin gingival phenotype, inadequate plaque control, traumatic toothbrushing, or excessive orthodontic tooth movement may increase the risk of soft tissue instability and GR [75,76].

### 5.2. Phenotype-Related Risk Stratification Before Orthodontic Treatment

Periodontal risk assessment should be incorporated into orthodontic treatment planning, particularly in patients presenting with thin gingival tissues, reduced KTW, limited facial alveolar bone, pre-existing dehiscences, unfavorable root position, poor plaque control, smoking exposure, or systemic conditions affecting bone metabolism. Identification of these factors before treatment may support appropriate modification of orthodontic biomechanics and the intensity of periodontal monitoring.

## 6. Cone-Beam Computed Tomography in Orthodontic–Periodontal Risk Assessment

### 6.1. CBCT Imaging Principles and Clinical Applications

CBCT provides three-dimensional visualization of dental and craniofacial structures through volumetric reconstruction of multiple two-dimensional X-ray projections acquired during rotational scanning. Unlike conventional two-dimensional radiography, CBCT eliminates image superimposition and geometric distortion, enabling precise assessment of teeth, roots, and alveolar bone morphology [77]. Owing to its increasing availability, CBCT has become a valuable three-dimensional imaging modality in implant dentistry, orthodontics, endodontics, and oral surgery when appropriately indicated [78]. In orthodontic-periodontal treatment planning, however, its principal value lies in biologically guided risk assessment by visualizing the alveolar housing and identifying anatomical limitations before orthodontic tooth movement [79].

### 6.2. Evaluation of Alveolar Bone Morphology

A major advantage of CBCT is the ability to evaluate the three-dimensional morphology of the alveolar process and determine the biologic boundaries of safe tooth movement. CBCT enables assessment of cortical and cancellous bone thickness, alveolar crest morphology, root inclination, root position within the alveolar housing, and pre-existing osseous defects that may remain undetectable clinically or on conventional radiographs [80]. Such information is particularly valuable in patients with a thin periodontal phenotype, severe crowding, planned arch expansion or incisor proclination, and pre-existing GR, in whom exceeding the adaptive capacity of the cortical plate may increase the risk of periodontal complications [51,81,82,83].

Importantly, roots may already be positioned close to the facial cortical plate before treatment begins. CBCT therefore allows clinicians to identify situations in which ideal orthodontic objectives conflict with the biologic limitations of the alveolar housing, facilitating individualized and phenotype-driven treatment planning.

### 6.3. Detection of Dehiscence and Fenestration

CBCT has substantially improved the detection of alveolar dehiscences and fenestrations, defects that frequently remain clinically undetectable before orthodontic treatment [84]. Identification of these defects is clinically relevant because orthodontic movement involving compromised cortical support may further increase the risk of GR and periodontal instability [76].

Clinical evidence also suggests that pre-existing alveolar defects reduce the rate of orthodontic tooth movement, from approximately 1.17 mm/month in intact alveolar bone to 0.62 mm/month in defect sites, potentially resulting in asymmetric tooth movement, altered force distribution, and an increased risk of external apical root resorption [85].

Several CBCT-based studies have reported increased prevalence of dehiscence-like defects following orthodontic treatment, particularly after excessive proclination or expansion mechanics [86,87]. However, these observations should be interpreted cautiously because very thin cortical plates frequently approach the spatial resolution limits of CBCT, potentially leading to overestimation of defect prevalence. Consequently, CBCT findings should always be interpreted together with clinical examination, periodontal phenotype assessment, and planned orthodontic biomechanics.

### 6.4. Limitations and Accuracy of CBCT in Periodontal Assessment

Despite its significant diagnostic value, CBCT has important limitations. Accurate visualization of thin cortical plates and small osseous defects remains influenced by voxel size, partial-volume averaging, metallic artifacts, and patient movement [87,88,89,90].

Particularly in the anterior region, facial cortical bone may be thinner than the spatial resolution of CBCT systems, resulting in false-positive diagnosis of dehiscence or fenestration [89,91]. Therefore, CBCT should not be regarded as a direct histologic representation of alveolar anatomy but rather as a complementary diagnostic tool requiring interpretation within the context of clinical findings.

Another important limitation is the inability of CBCT to accurately visualize gingival tissues and other soft tissue characteristics. Gingival thickness, keratinized tissue width, and soft tissue contours cannot be reliably assessed using CBCT alone, providing the rationale for integrating CBCT with intraoral optical scanning to achieve simultaneous visualization of hard and soft tissues [92].

### 6.5. Radiation Exposure and Ethical Considerations

Although CBCT exposes patients to substantially lower radiation doses than conventional medical computed tomography, radiation exposure remains higher than with conventional two-dimensional dental imaging. Consequently, routine CBCT examination for all orthodontic patients is not justified.

Current recommendations advocate an indication-based approach, in which CBCT should be prescribed only when additional three-dimensional information is expected to influence diagnosis, treatment planning, or patient safety [93]. From a periodontal perspective, CBCT is particularly valuable in patients presenting with a thin periodontal phenotype, severe crowding, planned arch expansion or incisor proclination, suspected dehiscences, pre-existing GR, or anatomically compromised alveolar housing.

CBCT acquisition should follow the principles of radiation protection, particularly ALARA (As Low As Reasonably Achievable) and the more recent ALADAIP (As Low As Diagnostically Acceptable, being Indication-oriented and Patient-specific), with optimization of field of view, voxel size, and exposure parameters according to the specific diagnostic objective [94]. Ultimately, CBCT should be prescribed only when the expected diagnostic benefits outweigh the associated biologic risks, supporting a personalized and biologically justified approach to orthodontic treatment planning.

## 7. Digital Dentistry and STL-CBCT Fusion

### 7.1. Intraoral Optical Scanning in Periodontal Assessment

Intraoral optical scanners (IOSs) project structured light onto the teeth and surrounding soft tissues and capture the reflected signal using optical sensors. Dedicated software reconstructs these images using triangulation or confocal imaging algorithms to generate highly accurate three-dimensional (3D) digital models of the dentogingival complex [95]. Compared with conventional impressions, IOSs improve patient comfort, reduce chairside time, and eliminate inaccuracies associated with impression material distortion and gypsum expansion or shrinkage [96].

Digital intraoral scanning has become an integral component of prosthodontics, implant dentistry, orthodontics, and periodontology. High-resolution 3D models enable quantitative assessment of gingival morphology, soft-tissue volume, periodontal phenotype, GR dimensions, papillary height, keratinized tissue width, and gingival zenith position, while supporting longitudinal monitoring of periodontal disease progression and treatment outcomes [97,98]. Several studies have demonstrated excellent reproducibility and high accuracy of IOS for measuring gingival dimensions and recession defects, although accuracy decreases in areas covered by mobile mucosa because of tissue displacement during image acquisition [99,100].

Beyond their diagnostic value, IOSs facilitate digital treatment planning, virtual diagnostic wax-ups, interdisciplinary communication, and remote monitoring of periodontal and orthodontic patients. Nevertheless, intraoral scanning does not replace conventional periodontal examination, as probing pocket depth, bleeding on probing, and plaque accumulation still require direct clinical assessment [101].

### 7.2. STL-CBCT Fusion Protocols

The integration of intraoral optical scans (STL files) with CBCT (DICOM files) represents one of the most important advances in digital dentistry. In this workflow, CBCT provides three-dimensional visualization of tooth roots, alveolar bone, and adjacent anatomical structures, whereas the intraoral scan accurately captures the external morphology of teeth and gingival tissues. Dedicated software registers both datasets using surface-matching algorithms, most commonly the iterative closest point (ICP) algorithm, creating a unified three-dimensional model that combines precise hard- and soft-tissue information [102].

The accuracy of STL–CBCT fusion has been shown to be high, even in the presence of metallic restorations or imaging artifacts [102,103]. The resulting integrated models enable simultaneous evaluation of root position, alveolar bone morphology, gingival thickness, soft-tissue contours, and periodontal phenotype, thereby substantially improving diagnosis, orthodontic treatment planning, and longitudinal assessment of periodontal changes during tooth movement [104].

Integrated STL-CBCT models enable simultaneous evaluation of tooth position, alveolar housing, and soft-tissue morphology, thereby improving visualization of patient-specific anatomical limitations relevant to orthodontic treatment planning.

## 8. Artificial Intelligence and Machine Learning in Orthodontic Risk Prediction

### 8.1. Artificial Intelligence as an Integrative Platform

Artificial intelligence (AI) has rapidly evolved from a technology primarily focused on image recognition into an integrative platform capable of supporting precision medicine through the analysis of heterogeneous biological datasets. Machine learning (ML) and deep learning (DL) algorithms can simultaneously process clinical records, three-dimensional imaging, periodontal phenotype, and molecular biomarkers, enabling prediction models that extend beyond conventional anatomical assessment and incorporate patient-specific biological variability [105,106].

In orthodontics, this capability is particularly important because periodontal complications arise from complex interactions among biomechanics, alveolar bone morphology, inflammatory activity, tissue phenotype, and individual adaptive capacity. Consequently, prediction models based solely on radiographic measurements or planned tooth movement may fail to capture the multifactorial nature of periodontal tissue remodeling [8,10].

Emerging multimodal AI approaches are being developed to integrate CBCT-derived alveolar bone morphology, intraoral optical scans, STL-CBCT fusion models, periodontal phenotype, and clinical parameters into unified prediction models. By simultaneously evaluating gingival thickness, keratinized tissue width, alveolar bone thickness, root position within the alveolar housing, dehiscences, fenestrations, and planned orthodontic movements, such models may ultimately provide individualized estimates of periodontal risk before treatment initiation; however, their clinical applicability requires appropriate prospective and external validation [106,107].

Beyond morphometric analysis, the next generation of AI models is expected to incorporate omics-derived biomarkers, including proteomic, transcriptomic, metabolomic, and microRNA profiles obtained from GCF, saliva, and blood. Integration of these molecular signatures with imaging data may substantially improve prediction of GR, alveolar bone loss, root resorption, and other treatment-related complications by reflecting each patient’s individual biologic responsiveness to orthodontic forces [12,57,58].

Unlike conventional statistical approaches, deep neural networks continuously improve predictive performance through exposure to large longitudinal datasets. This capability creates the possibility of dynamic risk assessment, in which orthodontic biomechanics may be modified according to continuously changing biologic response rather than predetermined treatment protocols alone [105,108].

### 8.2. Automated Periodontal Phenotype Recognition

Assessment of the periodontal phenotype traditionally relies on clinical evaluation of gingival thickness, keratinized tissue width, and alveolar bone morphology, procedures that are time-consuming, operator-dependent, and susceptible to inter-examiner variability. Recent advances in AI have enabled automated segmentation and quantitative analysis of periodontal structures from CBCT scans, intraoral optical scans, and fused STL-CBCT datasets.

Deep learning algorithms are capable of automatically identifying anatomical landmarks and extracting quantitative parameters, including gingival thickness, alveolar bone thickness, root position, dehiscences, fenestrations, and CEJ-alveolar crest distance. Such objective measurements improve reproducibility and enable standardized periodontal phenotype classification while minimizing observer-related variability [107,108].

More importantly, emerging multimodal AI systems may integrate these morphometric parameters with clinical variables and molecular biomarkers, potentially enabling biologically informed prediction of periodontal complications. Following appropriate prospective and external validation, such models may help identify patients presenting with a thin periodontal phenotype, limited adaptive capacity, or increased molecular susceptibility to tissue breakdown before orthodontic treatment is initiated, thereby supporting individualized treatment planning and phenotype-driven biomechanics [12,56].

### 8.3. AI-Assisted Virtual Patients and Digital Twins

One of the most promising future applications of AI is the development of patient-specific digital twins. At present, however, digital twins in orthodontics remain a conceptual and investigational framework rather than a validated clinical tool. A digital twin represents a continuously updated virtual replica of the patient’s craniofacial complex generated through the integration of CBCT imaging, intraoral optical scanning, facial scanning, electronic health records, and molecular biomarkers [109,110].

Unlike conventional digital models, digital twins are envisioned to incorporate temporal biological information and therefore may enable simulation not only of tooth movement but also of periodontal tissue remodeling throughout treatment. Integration of mechanobiological pathways, inflammatory biomarkers, and patient-specific phenotype characteristics may allow prediction of GR, alveolar bone loss, orthodontically induced root resorption, and regenerative capacity before these events become clinically detectable [10,12].

Ultimately, digital twins may support truly personalized orthodontic treatment by recommending patient-specific force magnitude, biomechanics, treatment duration, monitoring intervals, and adjunctive periodontal interventions according to each individual’s biologic profile [106,109].

### 8.4. Explainable Artificial Intelligence

Despite remarkable progress, implementation of AI in orthodontics requires not only high predictive accuracy but also biological interpretability. Most currently available deep learning algorithms operate as black-box models, generating predictions without explaining the biological mechanisms underlying their decisions [105].

Consequently, increasing attention has been directed toward explainable artificial intelligence (XAI), which identifies the variables contributing most strongly to individual predictions. In periodontal risk assessment, XAI may reveal whether predicted GR is primarily driven by reduced alveolar bone thickness, thin gingival phenotype, unfavorable root position, elevated RANKL/OPG ratio, increased inflammatory activity, or specific proteomic signatures. Such biologically interpretable models may improve clinician confidence while simultaneously generating new hypotheses regarding the molecular mechanisms governing periodontal adaptation [106,111].

Overall, the principal future role of AI may lie in integrating clinical, imaging, and molecular information into patient-specific periodontal risk models [110,112]. However, prospective validation, external testing, and biological interpretability remain prerequisites for their routine clinical implementation.

## 9. Personalized and Interdisciplinary Treatment Planning

Orthodontic treatment planning in patients at increased periodontal risk should integrate the intended tooth movement with periodontal phenotype, alveolar bone morphology, root position, and patient-specific biological susceptibility. The primary objective should be to achieve the desired orthodontic correction while maintaining tooth roots within the adaptive capacity of the periodontal tissues [10,113].

The initial diagnostic step is to determine whether adequate space is available to achieve complete alignment without excessive expansion of the dental arches or labial displacement of the roots. In patients presenting with tooth-size–arch length discrepancy, treatment alternatives such as interproximal enamel reduction, extraction therapy, arch expansion, or limited alignment should be evaluated according to the available alveolar bone envelope rather than solely on the basis of occlusal considerations [114].

Particular attention should be paid to camouflage treatment of skeletal malocclusions, in which extensive proclination or retroclination of the incisors may displace roots beyond the cortical boundaries of the alveolar process. Such movements increase the likelihood of alveolar dehiscence, fenestration, and subsequent GR, particularly in patients with a thin periodontal phenotype and limited alveolar bone thickness [25,115,116].

CBCT has become an indispensable tool for evaluating the three-dimensional relationship between tooth roots and the surrounding alveolar housing. Precise visualization of root position relative to the cortical plates allows identification of pre-existing dehiscences, fenestrations, reduced facial bone thickness, and anatomical limitations that cannot be detected using conventional two-dimensional imaging. These findings should guide biomechanical planning with the aim of maintaining or relocating roots within the center of the alveolar envelope whenever possible [79,102,104].

Treatment biomechanics should likewise be adapted to the patient’s biologic phenotype. Individuals with thin gingival tissues and limited facial alveolar bone require lighter orthodontic forces, greater control of root torque, slower rates of tooth movement, and more frequent periodontal monitoring than patients with thick periodontal phenotype. Consequently, treatment planning should focus not only on the desired final tooth position but also on preserving periodontal health throughout active tooth movement [10,117].

In selected patients, periodontal phenotype modification should be considered before or during orthodontic treatment as part of an interdisciplinary risk-reduction strategy. Autogenous connective tissue grafts remain the reference standard for increasing gingival thickness and enhancing soft tissue stability [118]. Histologic studies have demonstrated that phenotype modification induces thickening of the epithelium, increased collagen density within the lamina propria, and improved structural characteristics of the gingival tissues, thereby increasing their resistance to mechanical and inflammatory challenges [117]. Emerging biomaterials, including collagen matrices, hyaluronic acid, and tissue-engineering approaches, may further expand therapeutic options for minimally invasive phenotype enhancement in appropriately selected patients [119,120]. Advanced imaging and, in the future, validated molecular and AI-based risk models may further support this individualized decision-making process.

Successful management of patients at increased periodontal risk therefore requires close collaboration among orthodontists, periodontists, radiologists, and restorative dentists. Such interdisciplinary decision-making allows treatment objectives to be adapted to the biologic limitations of the supporting tissues while optimizing both functional and esthetic outcomes. Rather than pursuing maximal tooth movement, contemporary orthodontic treatment should aim to achieve the optimal balance between occlusal correction, facial esthetics, and long-term periodontal stability.

## 10. Future Perspectives

### 10.1. Precision Dentistry and Predictive Orthodontics

**Figure 2:**
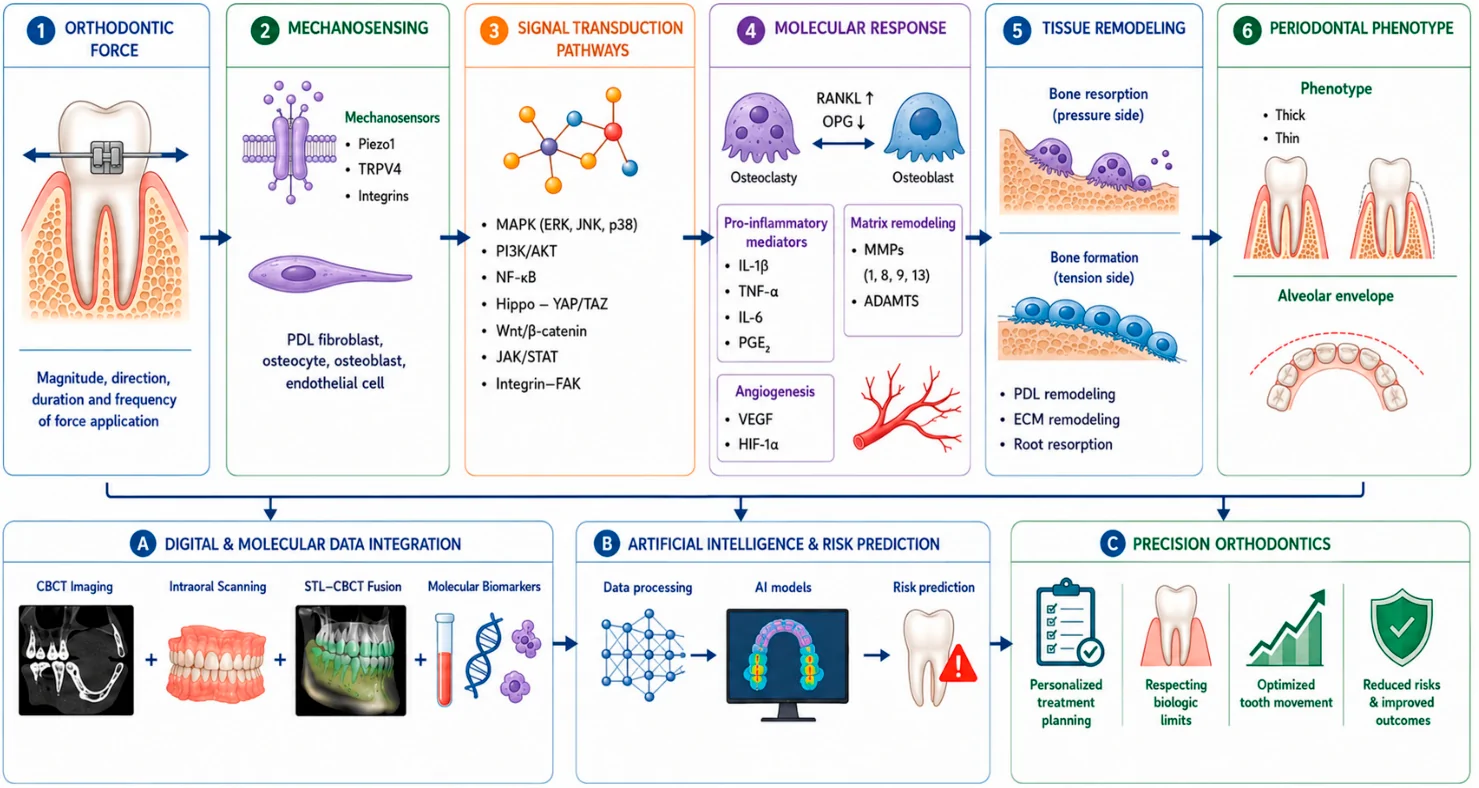
Biology-driven orthodontics: integration of mechanobiology, molecular signaling, periodontal phenotype, and artificial intelligence.

The next stage in biologically informed orthodontics is likely to involve dynamic integration of molecular, imaging, and longitudinal clinical data. Rather than relying solely on pretreatment anatomical assessment, future systems may continuously evaluate individual tissue responsiveness and adapt treatment accordingly. Several emerging approaches may contribute to this transition. Figure 2 schematically illustrates the integration of clinical, digital, and molecular data, whose analysis by artificial intelligence enables the development of an individualized treatment plan.

### 10.2. Multi-Omics and Biomarker Integration

Developments in proteomics are likely to facilitate the identification of patient-specific molecular signatures associated with bone turnover, tissue remodeling, and treatment response. The analysis of biomarkers in saliva, GCF, or blood could provide real-time information on an individual’s biological response to orthodontic forces, enabling dynamic adjustment of force magnitude and biomechanics throughout treatment. Although promising, multi-omics-based prediction currently remains a research-oriented approach, prospective clinical validation, standardized analytical workflows, and clinically meaningful predictive thresholds will be required before its incorporation into routine orthodontic risk assessment.

### 10.3. Future Directions in Personalized Periodontal Risk Assessment

Future research should focus on prospective multimodal models combining longitudinal clinical outcomes with quantitative imaging, periodontal phenotype, and molecular indicators of tissue responsiveness. Such datasets will be necessary to determine whether dynamic periodontal risk prediction can meaningfully improve orthodontic decision-making.

Advances in regenerative medicine suggest that MSCs may be capable of accelerating alveolar bone remodeling and improving the repair of root resorption defects following orthodontic treatment in preclinical models [121,122]. MSC-derived exosomes have demonstrated the ability to promote PDL regeneration and improve tooth stability predominantly in preclinical studies. These biological effects may provide a foundation for future strategies aimed at enhancing orthodontic anchorage and optimizing retention protocols following orthodontic treatment [123].

Hydrogels and collagen-based biomaterials have attracted considerable interest as localized drug delivery vehicles and regenerative scaffolds in orthodontics. Future applications may include the use of recombinant human collagen type I (rhCol I) as a bioactive extracellular matrix substitute to promote cell adhesion, regeneration of the PDL matrix and support periodontal tissue remodeling during and after orthodontic treatment [124,125].

At the molecular level, pharmacological modulation of bone remodeling offers the potential to selectively regulate the rate and direction of tooth movement. For example, the integrin antagonist cilengitide has demonstrated the ability to influence osteoclast adhesion and function, while MMP inhibitors may reduce excessive extracellular matrix degradation, thereby minimizing periodontal breakdown and orthodontically induced root resorption [126,127].

In parallel, targeted protein degradation technologies, including proteolysis-targeting chimeras (PROTACs), represent a novel experimental therapeutic platform capable of selectively eliminating key regulatory proteins involved in osteogenesis, osteoclastogenesis, and inflammatory signaling. Their application to orthodontic tissue remodeling remains hypothetical and has not been clinically validated. If successfully translated to orthodontics, this approach could provide greater precision in reducing unwanted bone resorption and improving the overall effectiveness of orthodontic tooth movement [128].

Moreover, the integration of molecular therapies with gene-based approaches, such as localized delivery of genes encoding osteogenic or osteoclast-regulating factors, RNA interference, CRISPR-mediated genome editing, and epigenetic modulation, represents a highly experimental research direction that may eventually enable individualized regulation of tissue remodeling according to each patient’s biological profile [129]. These strategies currently have no established clinical application in orthodontics.

The integration of molecular profiling with three-dimensional imaging, digital treatment planning, and artificial intelligence has the potential to establish a new paradigm of biologically informed precision orthodontics, in which therapeutic decisions are guided not only by anatomical and biomechanical considerations but also by each patient’s unique molecular phenotype. Such an approach could improve treatment efficiency, enhance long-term periodontal stability, and reduce the incidence of treatment-related complications. Table 4 summarizes potential future molecular strategies that may be incorporated into precision orthodontics.

## 11. Limitations of Current Evidence

### 11.1. Heterogeneity of Imaging Protocols

The interpretation of imaging studies investigating periodontal complications associated with orthodontic tooth movement is complicated by substantial methodological heterogeneity. CBCT acquisition parameters, including voxel size, field of view, exposure settings, reconstruction algorithms, and image processing protocols, differ considerably across studies, making direct comparisons difficult. Since the visualization of thin facial cortical bone is highly dependent on image resolution, differences in imaging protocols may influence the reported prevalence of dehiscence, fenestration, and alveolar bone loss. In addition, measurements are performed using different software platforms and reference landmarks, while image registration methods for CBCT and intraoral scans remain insufficiently standardized. These methodological differences reduce the comparability of published data and limit the development of robust quantitative thresholds for periodontal risk assessment.

### 11.2. Lack of Standardized Phenotype Assessment

Although the concept of the periodontal phenotype has become central to contemporary interdisciplinary treatment planning, no universally accepted assessment protocol has been established. Existing studies differ in the definition of GT, KTW, and alveolar bone morphotype, as well as in the diagnostic methods used to obtain these measurements. Clinical transparency techniques, transgingival probing, ultrasonography, CBCT, and digital intraoral scans have all been proposed for phenotype evaluation, yet each method measures different tissue characteristics and is associated with distinct advantages and limitations. Furthermore, studies continue to apply different phenotype classifications, ranging from binary thin and thick categories to more complex multidimensional systems. This methodological inconsistency limits reproducibility, complicates comparisons among studies, and hinders the development of universally applicable phenotype-based risk stratification systems.

### 11.3. Limited Prospective Clinical Evidence

Despite the increasing number of observational studies, high-quality prospective clinical evidence remains scarce. Most available investigations are retrospective, cross-sectional, or based on relatively small patient cohorts recruited from single institutions. Follow-up periods are frequently short, whereas patient populations differ substantially with respect to age, malocclusion type, periodontal status, orthodontic mechanics, and treatment objectives. Moreover, clinically relevant confounding variables, including oral hygiene, smoking, systemic diseases, medication use, inflammatory burden, and interindividual differences in bone metabolism, are often incompletely reported or insufficiently controlled. These limitations restrict the ability to establish causal relationships between orthodontic tooth movement, periodontal phenotype, and the subsequent development of periodontal complications.

### 11.4. External Validation of Artificial Intelligence Models

Artificial intelligence has shown promising results in image segmentation, automated phenotype recognition, and prediction of orthodontic treatment outcomes. However, most published algorithms have been developed using retrospective datasets obtained from single institutions and evaluated primarily through internal validation procedures. Variability in CBCT devices, imaging protocols, annotation strategies, patient demographics, and disease prevalence may substantially influence model performance when applied to independent populations. Furthermore, only a limited number of studies have performed external multicenter validation or followed current recommendations for the transparent reporting of prediction models. Consequently, the clinical generalizability and robustness of existing AI systems remain uncertain, limiting their implementation in routine orthodontic and periodontal practice.

### 11.5. Limited Integration of Molecular, Imaging, and Clinical Data

An important limitation of the current evidence is that molecular biology, imaging, and clinical diagnostics continue to evolve largely as independent research fields. Studies investigating inflammatory mediators, mechanotransduction pathways, or molecular biomarkers are rarely linked with quantitative imaging of the periodontal phenotype or with longitudinal clinical outcomes. Likewise, artificial intelligence models are predominantly trained using radiographic or photographic datasets, whereas biological variables reflecting individual tissue responsiveness are seldom incorporated into predictive algorithms. As a result, currently available risk assessment models capture only selected aspects of periodontal susceptibility and fail to reflect the complex biological processes underlying orthodontic tissue remodeling. Future research should focus on prospective multimodal studies integrating molecular biomarkers, periodontal phenotype, three-dimensional imaging, clinical parameters, and longitudinal treatment outcomes. Such datasets may provide the foundation for biologically informed prediction models capable of supporting truly personalized orthodontic treatment planning.

## 12. Conclusions

Orthodontic tooth movement is a mechanobiological process in which periodontal outcomes reflect the interaction between applied biomechanics and the patient-specific adaptive capacity of the supporting tissues. Periodontal phenotype, alveolar bone morphology, inflammatory and mechanotransduction pathways, and systemic factors contribute to the considerable interindividual variability observed during treatment.

Periodontal complications, including GR and alveolar bone loss, should therefore not be attributed to tooth movement alone. Pretreatment assessment of periodontal phenotype, root position, and the alveolar housing may help identify anatomical vulnerability and support appropriate modification of orthodontic biomechanics and periodontal monitoring.

Molecular biomarkers, multimodal imaging, and artificial intelligence may further improve individualized risk assessment, but these approaches remain largely investigational. Prospective multimodal studies and external validation are required before biology-driven predictive models can become established components of routine precision orthodontics.

Several limitations should be considered when interpreting the conclusions of this review. As a narrative review, the selection and synthesis of the literature were not based on a formal systematic-review protocol and may therefore be subject to selection bias. Moreover, several of the fields discussed, particularly molecular biomarkers, multimodal artificial intelligence, and precision-orthodontic applications, are evolving rapidly, and the available evidence is heterogeneous in terms of study design, methodological quality, and level of validation. Much of the detailed mechanistic evidence is derived from in vitro and preclinical studies, whereas prospective and externally validated clinical evidence remains comparatively limited. Consequently, some of the translational and predictive applications discussed herein should be regarded as emerging rather than established components of routine orthodontic practice.

## Figures and Tables

**Table 1:** Molecular pathways regulating orthodontic tooth movement.

Pathway	Main Molecules	Biological Function	Clinical Relevance	Level of Evidence
Piezo1	Piezo1	mechanosensing, Ca^2+^ influx	initiation of mechanotransduction	Predominantly preclinical/in vitro; limited human evidence
TRPV4	TRPV4	calcium signaling	adaptation to mechanical loading	predominantly preclinical/in vitro; limited human evidence
Integrin-FAK	Integrins, FAK	focal adhesions	force transmission	predominantly preclinical/in vitro; limited human evidence
Hippo	YAP/TAZ	osteogenesis, proliferation	alveolar remodeling	predominantly preclinical/in vitro; limited human evidence
Wnt	β-catenin, LRP5/6, SOST	bone formation	cortical adaptation	preclinical and human biomarker evidence
RANK pathway	RANK, RANKL, OPG	osteoclastogenesis	bone resorption	preclinical and human biomarker evidence
MAPK	ERK, JNK, p38	inflammatory signaling	tissue remodeling	preclinical and human biomarker evidence
PI3K/AKT	AKT	cell survival	osteoblast differentiation	predominantly preclinical/in vitro
Notch	Notch	stem cell differentiation	tissue homeostasis	predominantly preclinical/in vitro

**Table 2:** Molecular biomarkers associated with periodontal remodeling during orthodontic tooth movement.

Biomarker	Source	Function	Clinical Significance	Evidence Level
IL-1β	GCF	inflammation	increased remodeling	human clinical biomarker evidence
TNF-α	GCF	osteoclast activation	bone loss	human clinical biomarker evidence
IL-6	GCF	inflammation	remodeling activity	human clinical biomarker evidence
PGE2	GCF	bone resorption	orthodontic tooth movement rate	human clinical biomarker evidence
RANKL	GCF	osteoclastogenesis	alveolar resorption	human clinical biomarker evidence
OPG	GCF	inhibits osteoclasts	protective marker	human clinical biomarker evidence
MMP-1	GCF	collagen degradation	ECM remodeling	human clinical biomarker evidence
MMP-8	GCF	collagen degradation	tissue destruction	human clinical biomarker evidence
MMP-9	GCF	matrix degradation	periodontal breakdown	human clinical biomarker evidence
ALP	GCF	osteogenesis	bone formation	human clinical biomarker evidence
Runx2	cells	osteoblast differentiation	bone apposition	predominantly preclinical/cellular evidence
VEGF	tissues	angiogenesis	vascular adaptation	preclinical + limited human evidence
miRNAs	saliva/GCF	gene regulation	future biomarker	emerging human/preclinical evidence
Exosomes	EVs	regeneration	future therapy	predominantly preclinical evidence

GCF—gingival crevicular fluid; EVs—extracellular vesicles; ECM—extracellular matrix.

**Table 3:** Mechanobiological events during orthodontic tooth movements.

Stage	Biological Event	Major Mediators
Immediate	mechanosensing	Piezo1, TRPV4
Early	inflammation	IL-1β, TNFα, IL-6
Early remodeling	osteoclastogenesis	RANKL
Late remodeling	osteogenesis	Runx2, BMP, Wnt
Tissue adaptation	ECM remodeling	MMPs
Homeostasis	bone stabilization	OPG

**Table 4:** Future molecular strategies for precision orthodontics.

Technology	Mechanism	Potential Future Application	Current Translational Status
miRNA profiling	gene regulation	risk prediction	investigational; requires prospective clinical validation
Proteomics	molecular signature	personalized diagnosis	early human evidence; not validated for routine personalized prediction
Exosomes	regeneration	accelerated healing	predominantly preclinical; investigational
MSC therapy	osteogenesis	tissue regeneration	preclinical in orthodontic applications; investigational
Hydrogels	local delivery	targeted therapy	predominantly preclinical/experimental
AI	multimodal integration	precision treatment	emerging; requires prospective and external validation
Digital twins	virtual simulation	individualized planning	conceptual/investigational; no established routine clinical application

## Data Availability

No new data were created or analyzed in this study. Data sharing is not applicable to this article.
